# DOES MOTIVATION PROMOTE PHYSICAL ACTIVITY IN OLDER ADULTS WITH CHRONIC MUSCULOSKELETAL PAIN?

**DOI:** 10.1093/geroni/igac059.3090

**Published:** 2022-12-20

**Authors:** Kailyn Witonsky, Xiaonan Zhu, Andrea Rosso, Caterina Rosano

**Affiliations:** University of Pittsburgh, Pittsburgh, Pennsylvania, United States; University of Pittsburgh, Pittsburgh, Pennsylvania, United States; University of Pittsburgh, Pittsburgh, Pennsylvania, United States; University of Pittsburgh, Pittsburgh, Pennsylvania, United States

## Abstract

Chronic musculoskeletal pain is common and limits physical activity in older adults. Understanding why some older adults remain physically active despite chronic musculoskeletal pain, “pain resiliency”, may offer insights to promote physical activity. We investigated the cross-sectional association of three indicators of motivation or mood with physical activity in adults over the age of 65 with chronic musculoskeletal pain from the Cardiovascular Health and Cognition Study. Of 2,703 participants with brain scans, 919 (34%) reported at least one month of either foot, knee, hip, or back pain and 47% of those with pain had moderate to high levels of self-reported physical activity, defined as “pain resilient”. Indicators of motivation included: (1) self-reported motivation: a composite score of perceived effort, difficulty getting going in the morning, and difficulty concentrating, (2) social network score from the Lubben Social Network scale, and (3) a composite depression scale. Separate multivariable linear models estimated the association between physical activity and each indicator, controlling for gender, race, BMI, age, gait speed, number of medications, number of pain sites, cognitive function, as measured by the digit substitution symbol test, and brain integrity, as measured by level of white matter hyperintensities. Higher self-reported motivation (β=-0.11, p=0.02) and larger social networks (β=0.03, p=0.01) were associated with higher levels of physical activity. Better mood was not associated with higher levels of physical activity (β=-0.017, p=0.34). Albeit based on cross-sectional self-reported measures, our research suggests the need for further research investigating psychosocial contributions to pain resilience.

